# Identification of resistance loci in Chinese and Canadian canola/rapeseed varieties against *Leptosphaeria maculans* based on genome-wide association studies

**DOI:** 10.1186/s12864-020-06893-4

**Published:** 2020-07-21

**Authors:** Fuyou Fu, Xuehua Zhang, Fei Liu, Gary Peng, Fengqun Yu, Dilantha Fernando

**Affiliations:** 1 Department of Plant Science, University of Manitoba, Winnipeg, MB R3T 2N2 Canada; 2 Saskatoon Research Centre, Agriculture and Agri-Food Canada, 107 Science Place, Saskatoon, Saskatchewan S7N 0X2 Canada

**Keywords:** Genotyping-by-sequencing (GBS), Genome-wide association study (GWAS), Resistance gene analogues (RGAs), *Brassica napus*, *Leptosphaeria maculans*, Blackleg, Phoma stem canker

## Abstract

**Background:**

The fungal pathogen *Leptosphaeria maculans* (*Lm*). causes blackleg disease on canola/rapeseed in many parts of the world. It is important to use resistant cultivars to manage the disease and minimize yield losses. In this study, twenty-two *Lm* isolates were used to identify resistance genes in a collection of 243 canola/rapeseed (*Brassica napus* L.) accessions from Canada and China. These *Lm* isolates carry different compliments of avirulence genes, and the investigation was based on a genome-wide association study (GWAS) and genotype-by-sequencing (GBS).

**Results:**

Using the CROP-SNP pipeline, a total of 81,471 variants, including 78,632 SNPs and 2839 InDels, were identified. The GWAS was performed using TASSEL 5.0 with GLM + Q model. Thirty-two and 13 SNPs were identified from the Canadian and Chinese accessions, respectively, tightly associated with blackleg resistance with *P* values < 1 × 10^− 4^. These SNP loci were distributed on chromosomes A03, A05, A08, A09, C01, C04, C05, and C07, with the majority of them on A08 followed by A09 and A03. The significant SNPs identified on A08 were all located in a 2010-kb region and associated with resistance to 12 of the 22 *Lm* isolates. Furthermore, 25 resistance gene analogues (RGAs) were identified in these regions, including two nucleotide binding site (NBS) domain proteins, fourteen RLKs, three RLPs and six TM-CCs. These RGAs can be the potential candidate genes for blackleg resistance.

**Conclusion:**

This study provides insights into potentially new genomic regions for discovery of additional blackleg resistance genes. The identified regions associated with blackleg resistance in the germplasm collection may also contribute directly to the development of canola varieties with novel resistance genes against blackleg of canola.

## Background

*Brassica napus* L. (AACC, 2n = 38) is one of the important crop used for oil, vegetable, fodder, and bio-fuel [1–4]. Blackleg (Phoma stem canker), caused by the fungal pathogen *Leptosphaeria maculans* is one of the major diseases on canola and rapeseed (*B. napus*) in many parts of the world. Prior research has determined that *L. maculans* can cause severe infection on a susceptible canola cultivar and reduce the seed yield by more than 50% [5]. Improving resistance to *L. maculans* is one of the major objectives in canola breeding programs in Canada, especially due to the fact that the disease is difficult to control with fungicides [6].

Two types of resistance to *L. maculans* have been reported in *B. napus* [7]; qualitative resistance mediated by an effector-triggered immunity (ETI) mechanism with specialized interactions between a race-specific R protein and corresponding avirulent (Avr) protein. Currently, R gene resistance is the most effective method against blackleg in canola, and 19 R genes have been identified in *B. rapa*, *B. juncea*, *B. napus*, and *B. nigra* [8–16]. However, the pathogen populations have also been rapidly evolving, with high selection pressure, due to broad utilization of single-gene resistance in commercial fields. This results in rapid erosion of resistance as the pathogen population evolves. In Australia, *LepR3* resistance derived from *B. rapa* subsp. *sylvestris* was overcome within 3 years after the commercial release of the cultivars [17]. Rouxel et al. [18] also reported that the frequency of *AvrLm1* was rapidly decreased due to the increased commercial use of the *Rlm1* resistance gene in France. In western Canada, a few studies have revealed that the structure of *Lm* population has been shifting over the past decade; PG2 was classified as the primary pathogenicity group (PG) of *Lm* collected between 1984 and 2000 [19, 20], but PG3 and PGT emerged from *Lm* isolates collected between 1998 and 2004. Liban et al. provided further evidence on a shift in avirulence allele frequencies in *Lm* isolates collected in 2010 and 2011 [21]. Zhang et al. reported that*Rlm3* resistance had been broken down because *AvrLm3* was no longer a predominant avirulence effector in western Canada [22]. These studies indicate that the *Lm* population has evolved with the selection pressure from resistant canola varieties which carry a limited number of resistance genes; a single R gene will unlikely provide durable resistance against highly diverse *Lm* races in western Canada. Continued efforts to identify novel resistance loci from canola or rapeseed germplasm can aid in blackleg resistance breeding by providing new resistance sources for disease management. Another type of resistance is quantitative resistance (QR, race non-specific), which is conferred typically by pattern-triggered immunity (PTI) mechanisms in conjunction with pathogen associated molecular patterns [23, 24]. Several QR loci have been identified for resistance to *Lm* using traditional QTL and/or genome wide association studies (GWAS) approaches on the genetic/physical maps of *B napus* [25–35]. Darmor-bzh/Yudal (DY) [25, 26, 34, 35], Topas/AG-Castle (TC) and Topas/AV-Sapphire (TS) [26–28], Skipton/Ag-Spectrum (SASDH) [32] DH population and a few diverse panels [26, 30] of *B .napus* were employed to identify QR loci for blackleg resistance in canola. However, Canadian and Chinese canola germplasm has not been used to identify QR or QTL against the diverse Canadian *Lm* population.

China imports a substantial amount of canola/rapeseed seed, meal and oil from countries like Canada and Australia each year [36]. Since the pathogen *Lm* has not been reported in China, the inoculum on imported canola seeds is deemed a risk of introduction [37]. In fact, blackleg has been closely monitored in China [38], with disease surveys conducted frequently. So far the results have indicated that blackleg in China is caused exclusively by *L. biglobosa* [36–40], a less aggressive species of *Leptosphaeria*. It may be useful to minimize *Lm* contamination of seed/dockage for export to China [41], identification of resistance genes in Chinese varieties will also assist in Chinese scientists to develop effective resistant cultivars quickly once *Lm* is found in the country.

Association mapping (AM) has been used as a powerful tool to overcome some of the limitations of bi-parental mapping for QTL discoveries [42, 43]; it can reduce the time lag between QTL discoveries and marker-assisted selection (MAS) [44], especially with a huge number of SNP markers identified with next generation sequencing. AM (also referred to as linkage disequilibrium mapping) is the non-random association between molecular markers and a phenotypic trait in a collection of genetically diverse germplasm [45]. Historic recombination between a marker and the locus associated with the trait of interest is exploited to uncover significant correlations between markers and phenotypic traits. Therefore, genome-wide association studies offers scanning of marker-trait associations using moderate marker densities, whereas fine mapping is achieved through subsequent mapping with higher density markers [43, 46]. AM has been successfully employed to map complex traits in plants, including disease resistance, such as resistance to *Pseudomonas syringae* in *Arabidopsis thaliana* [47], *Stagonospora nodorum* blotch in wheat [48], stem rust resistance in barley [49], bymovirus in barley [50] and fusarium head blight in barley [51]. In a well-designed association mapping study, newly discovered QTLs could immediately be used for MAS. In *B. napus,* AM mapping of complex traits has focused mostly on oil [52, 53] and glucosinolate [54] content, phenolic compounds [55] and agronomic traits [52, 56]. Jestin et al. [30] used AM to map QTLs for resistance to blackleg caused by *Lm* in *B. napus*. AM with genotyping-by-sequencing (GBS) has been widely used to map QTLs in crops. GBS can generate millions of SNPs using next generation sequencing technologies with low cost [57, 58]. We have successfully mapped three QTL for clubroot resistance to six pathotypes of *Plasmodiophora brassicae* in *Brassica rapa* with GBS [59]. In this study, new resistance accessions were identified during screening for blackleg resistance in a world collection of 243 *B. napus*. Meanwhile, potential new blackleg resistance loci were found with genome-wide association study using GBS.

## Results

### Genetic variation in resistance to *L. maculans*

**Fig. 1:**
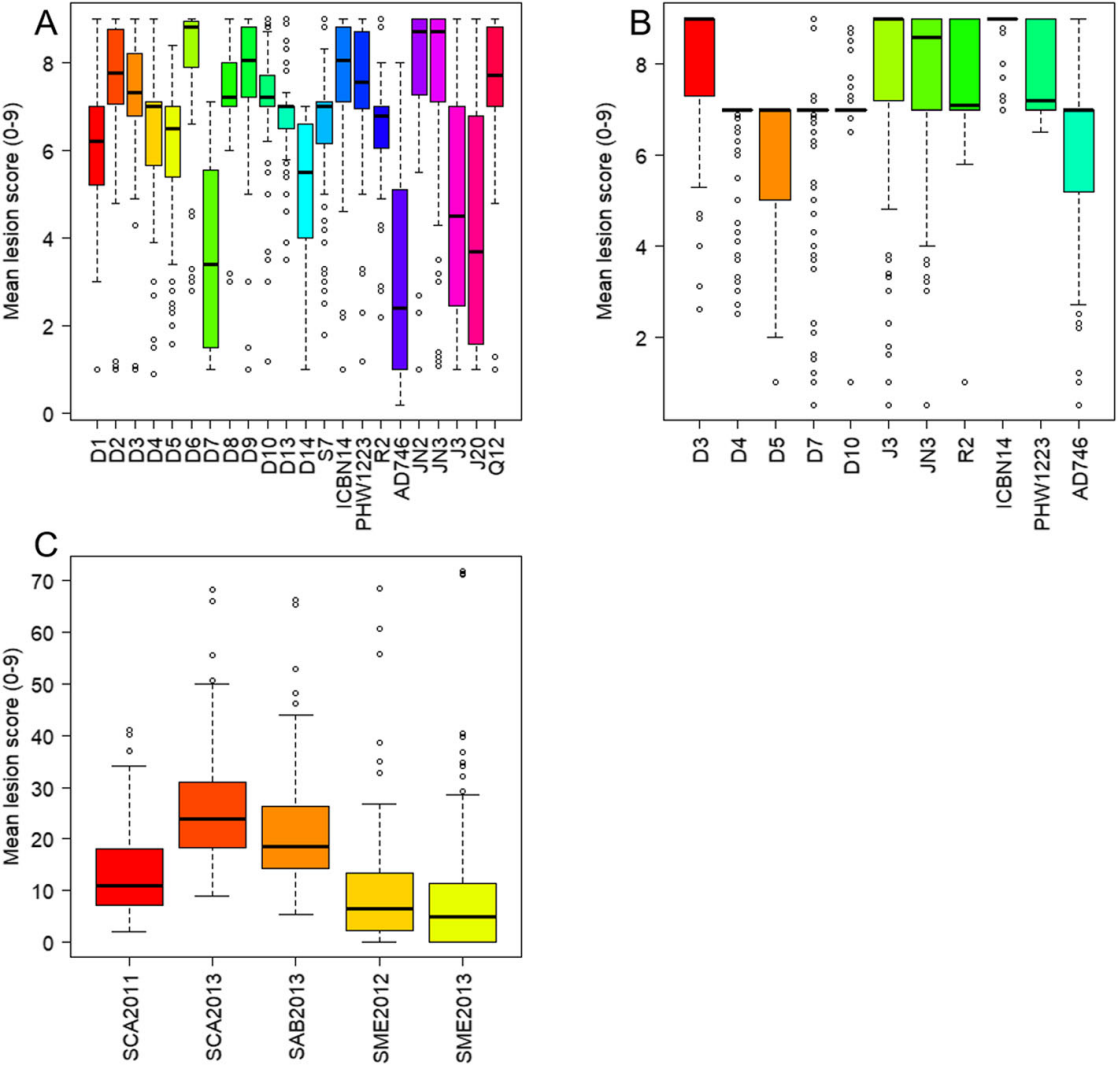
Phenotypic variation for reistance to L. maculans. **a** Box-plots showing seeding phenotypic variation to 22 L. maculans isolates in 93 Canadian accessions. **b** Box-plots showing seeding phenotypic variation to 12 L. maculans isolates in 150 Chinese accessions. **c** Box-plots showing phenotypic variation in 150 Chinese accessions with five field trials

Total of 22 *Lm* isolates were employed to assess the resistance of 243 canola accessions at the seedling (cotyledon) stage. The Avr-gene compliments of the isolates have been reported previously [22, 60] and are listed in Table S1. Both Canadian accessionsand Chinese accessions presented quantitive trait characterization among of 22 Lm isolates and five field trials (Fig. 1). Extensive variation was observed among the accessionstested for the resistance; the lesion score ranged from 1 to 9 among both Canadian and Chinese accessions on cotyledons, with the majority of them being susceptible (lesion scores > 5) to all the *Lm* isolates, especially those from China (Fig. 1a and b).

### Identification of DNA variants

**Fig. 2:**
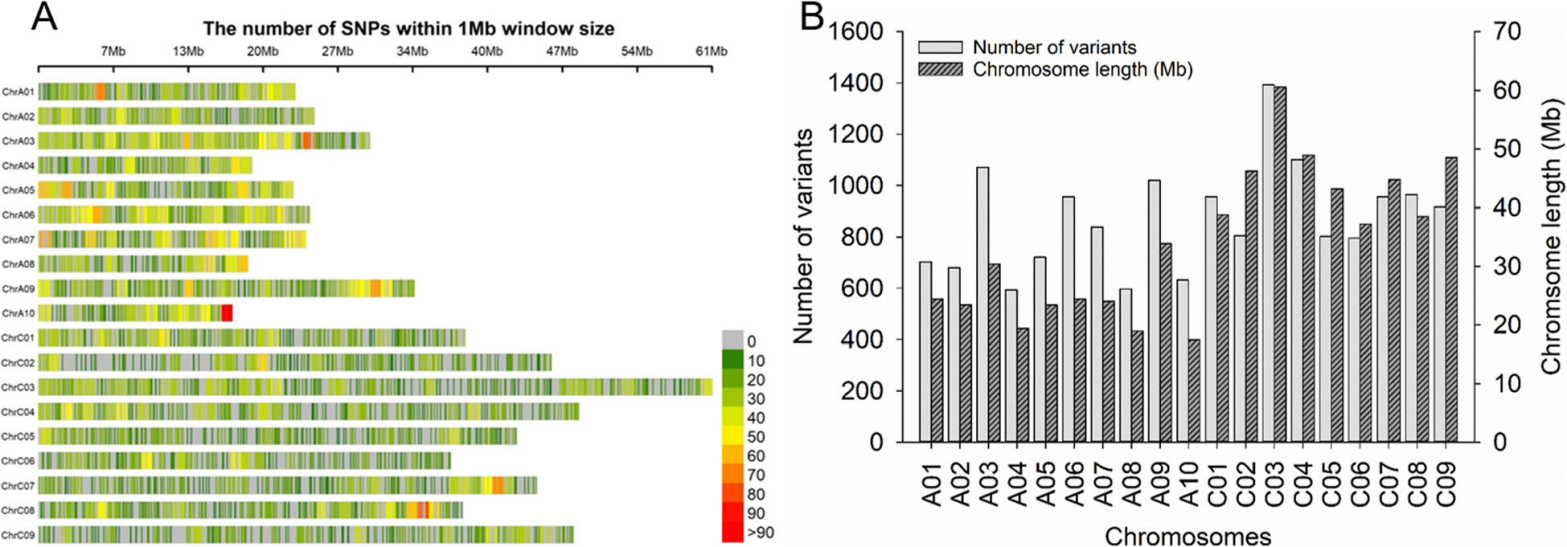
The distrubition of variants (SNPs and InDels) each chromosome based on *B. napus* reference genome. **a** Number of SNPs within 1 Mb windows size in each chromosome. **b** Number of plolymorphic variants (SNPs and InDels) in each chromosome

A total of 243 *B. napus* accessions collected from China and Canada [22, 60] were analysed with the GBS method on an Illumina HiSeq 2500 (PE125) system (Table S2), with a total of 1054 million reads generated. Based on the output of GBS-SNP-CROP pipeline V3.0 [61], a total of 81,471 high-quality DNA variants (78,632 SNPs and 2839 InDels) were identified using the *B. napus* reference genome [62] under the parameters described in Methods, (Fig. 2a). InDels were not considered for GWAS in this study. The number of variants was significantly correlated (*P* = 0.000574, Spearman correlation) with the length of chromosomes (Fig. 2b). All variants were converted into plink and hmnp in Tassel format using the scripts of the GBS-SNP-CROP pipeline v3.0 [61] for further GWAS.

### GWAS identified resistance loci to *L. maculans*

A total of 16,503 SNP loci were obtained after filtering at MAF > 5% and missing data < 10%, and then used for GWAS using general linear model (GLM) with two methods GLM + Principal component analysis (PCA) and GLM + (Q). The results fromGLM+PCA GLM + Q were very similar. Additionly, no difference was found in the significant SNP loci identified from orginal phenotypic data and from nomoralized data (data not shown). Hence, in this study, GLM + *Q* module with original phenotypical data was performed as identify the significantly associated SNP loci.

GWAS identified loci for cotyledon resistance to *L. maculans* in Canadian canola accessions.

**Fig. 3:**
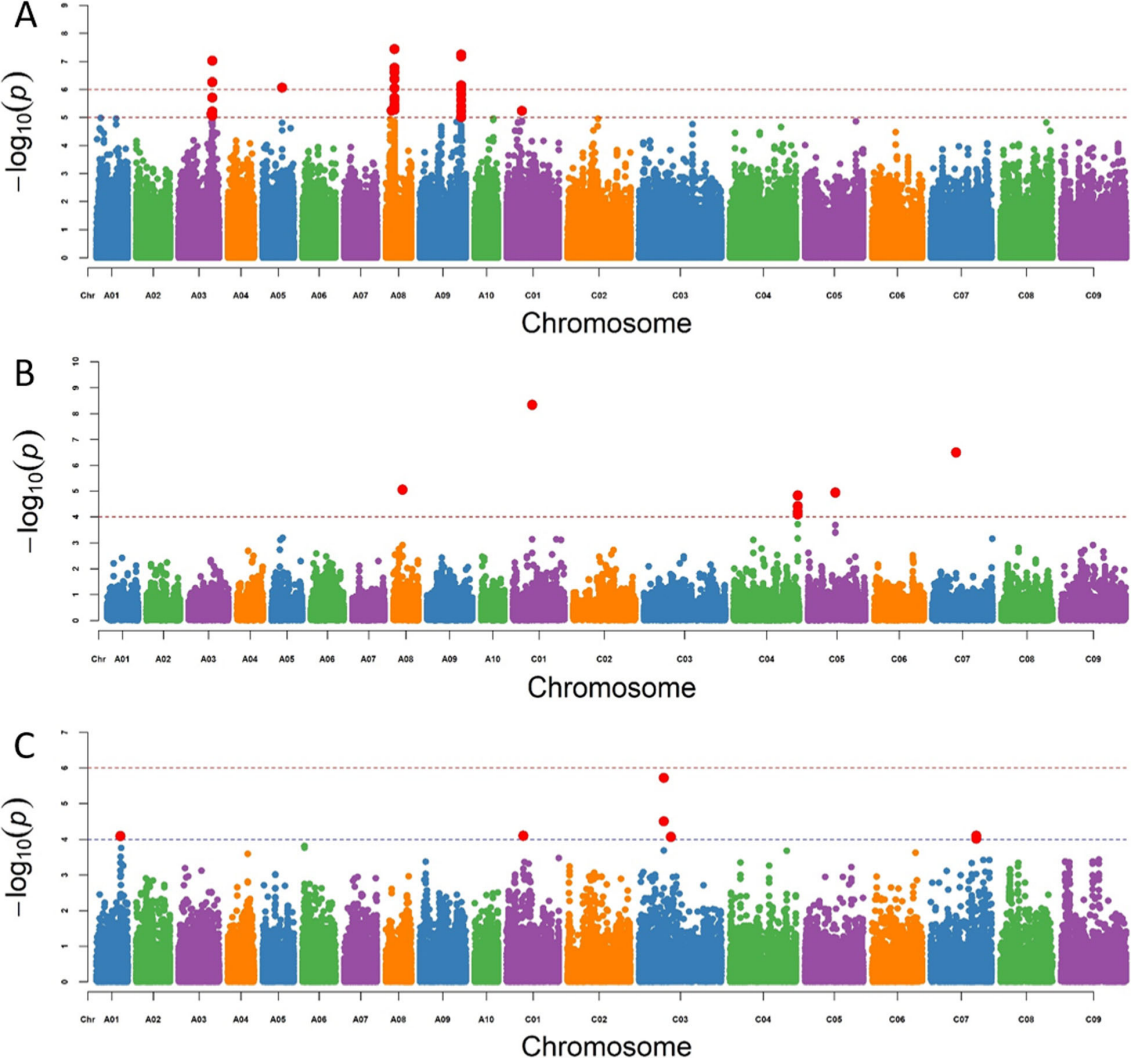
Manhattan plots showing the results of genome-wide association study for resistance to L. maculans with seedling and adult-plant stages in Canadian and Chinese accessions. Red dots present significant SNPs with *P* value < 1 × 10^− 5^ in Canadian germplasm and 1 × 10^− 4^ in Chinese germplasm. Dash line present the threshold values. **a** shows significant SNPs in 93 Canadian accessions. **b** shows significant SNPS in 150 Chinese accessions. **c** shows significant SNPs in Chinese accessions with five field trials

**Fig. 4:**
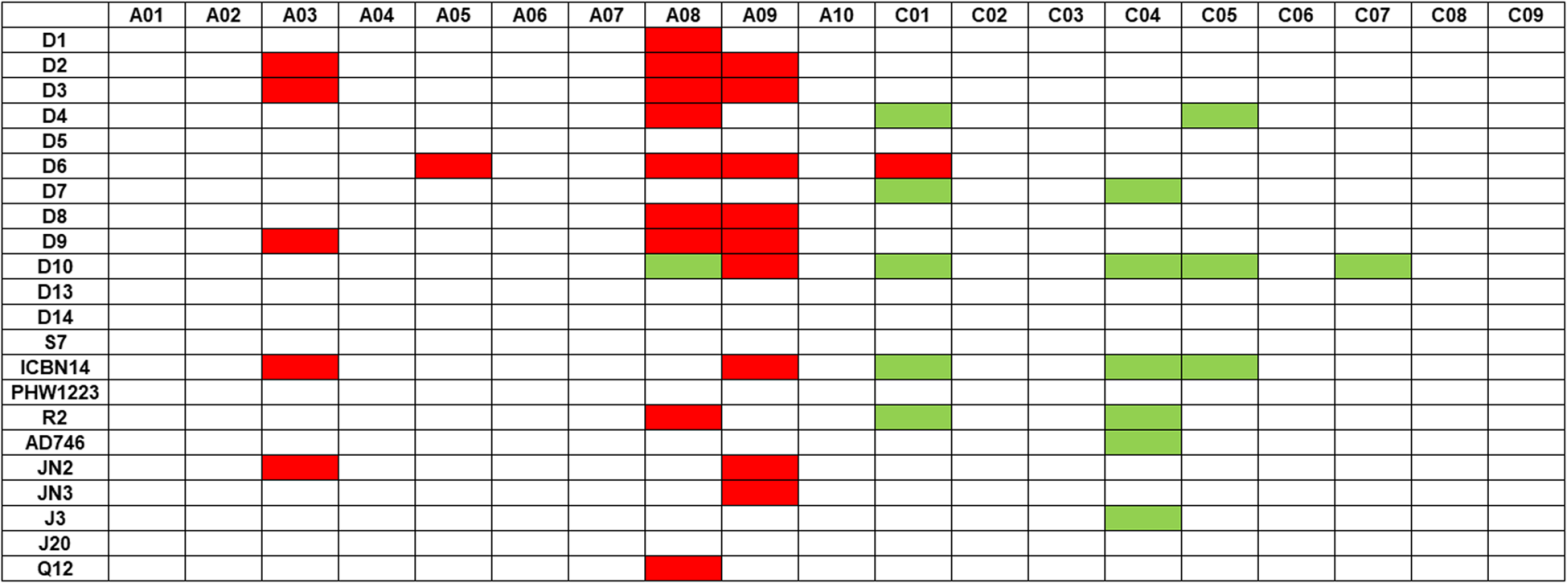
Summary of the resistance loci in Candian and Chinese accessions. Red box presents the loci in Canadian accessions. Green box presents the loci in Chinese accessions

Resistance loci in Canadian and Chinese canola accessions against *L. maculans* were dissected at both seedling and adult-plant stages using GWAS in TASSEL 5.0 with GLM + Q. A total of 111 significant loci were identified with 22 isolates in Canadian varieties, with 12.1–31.6% phenotypic variations explained by resistance loci (Table S2). These associated-resistance loci were mostly located on chromosomes A03, A08 and A09 (Fig. 3a and Fig. 4), with 30 of them identified repeatedly against multiple *Lm* isolates (Table S2). On chromosome A03, three SNPs (delimited with 24,158,622 | A > C, 24717825 | T > C, and 24,731,214 | C > T, respectively) were located within a 572-kb region. Four SNPs (6,430,433 | C > G, 6430461 | T > A, 6430502 | T > C, and 6,441,932 | G > T, respectively) were detected within an 11-kb region on chromosome A08. Two SNPs (30,423,813 | C > T and 30,423,849 | T > G) were found within a very narrow region (36 bp) on chromosome A09. No resistance loci were detected from these Canadian varieties at the seedling stage against 6 of the *Lm* isolates (D7, D13, D14, AD746, J3 and J20) at *P* < 10^− 4^.

-

A total of 57 significant SNP loci were identified against the 12 *Lm* isolates in Chinese canola accessions with 9.1–24.3% phenotypic variation in resistance (Table S3). These resistance-associated SNP loci were mostly located on chromosomes A08, C01, C04, C05 and C07 (Fig. 3b and Fig. 4), with 35 of them identified repeatedly against multiple isolates (Table S2). Most of theassociated SNPs were detected with isolate D10; 35 SNPs were located on chromosomes A08, C01, C04, C05 and C07.

On chromosome A08, 6 SNPs (6,986,581 | G > C, 6986629 | A > T, 6986688 | A > T, 6986854 | T > G, 6986868 | C > T, 6986893 | A > G) were identified within a narrow region (312 bp). On chromosome C01, there were also 6 SNPs (14,069,868 | C > T, 14069974 | T > C, 14087080 | A > T, 14087137 | A > G, 14090999 | G > A and 14,091,013 | A > C) that were detected in a 21-kb region. Four SNPs (47,531,363 | A > G, 47531439 | G > A, 47531442 | T > C and 47,531,461 | G > C) were found in a 98-bp region on chromosome C04. Twelve SNPs (20,249,568 | G > T, 20452502 | A > C, 20452533 | G > A, 20452554 | A > C, 20599157 | C > A, 20599238 | G > A, 21291258 | C > T, 21291283 | T > C, 21637008 | T > C, 21637016 | A > C, 21637090 | T > A, and 21,564,255 | A > G) were located in two regions (147 kb and 346 kb, respectively) on chromosome C05. Twelve SNPs (37,421,042 | T > C, 37421119 | C > A, 37421931 | G > C, 37583190 | T > A, 37583265 | C > T, 37583295 | T > G, 37583379 | T > C) were located within a 162-kb region on chromosome C07.

A total of 13 significantly-associated loci were identified based on the resistance rating in 5 field trials in western Canada, with 8.7–18.7% phenotypic variation in resistance (Table S4). These resistance loci were located on chromosomes A01 (17,731,177 | A > G), C01 (12,182,933 | A > G), C03 (18,318,846 | T > C and 23,396,866 | A > G) and C07 (33,405,386 | A > C) (Fig. 3c and Fig. 4).

### Potential resistance gene analogues against *L. maculans* in *B. napus*

**Fig. 5:**
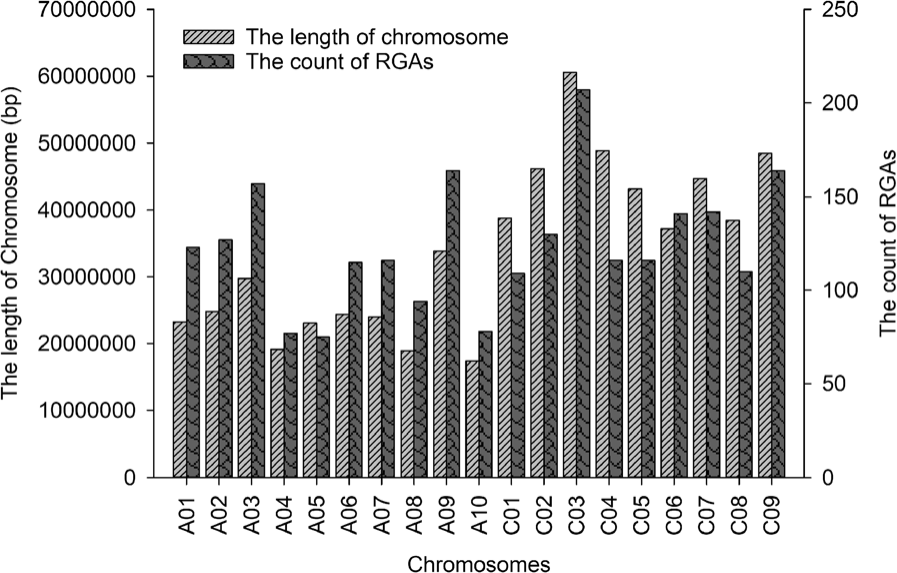
The distrubition of RGAs each chromosome based on *B. napus* reference genome

A total of 2821 RGA candidates were identified and classified into four major families based on the combinations of these RGA domains and motifs with the RGAugury pipeline [63] in the *B. napus* reference genome [62], including 627 NBS-encoding proteins, 1503 receptor-like kinases (RLKs), 276 receptor-like proteins (RLPs) and 415 transmembrane coiled-coil proteins (TM-CC) (Table S5). These RGAs were evenly distributed on 19 *B. napus* chromosomes (Fig. 5 and S1).

Twelve associated regions were identified within 200 kb from the most significant SNP loci associated with the resistance, including three, four and five regions in the cotyledon stage of Canadian and Chinese varieties and adult-plant stage of Chinese varieties, respectively (Table 1). Twenty-five RGAs were identified in these regions, including two NBS-domain proteins, fourteen RLKs, three RLPs and six TM-CCs.

**Table 1:** Potential resistance gene analogues against *L. maculans* in *B. napus*

Accessions	Chr	Position	candidate genes	Protein family
Canadian	A03	24,158,622	BnaA03g47360D	RLK
			BnaA03g47390D	TM-CC
			BnaA03g48410D	RLK
	A08	6,430,433	BnaA08g06440D	RLK
			BnaA08g07010D	TM-CC
	A09	30,423,813	BnaA09g44200D	RLK
			BnaA09g44590D	RLK
Chinese	C01	14,069,868	BnaC01g19470D	RLK
			BnaC01g20530D	TM-CC
	C04	47,531,363	BnaC04g49200D	RLK
			BnaC04g50000D	RLP
	C05	20,249,568	BnaC05g24620D	RLP
			BnaC05g26730D	RLK
	C07	37,421,042	BnaC07g34000D	NBS > TN
			BnaC07g35850D	RLK
Field	A01	17,731,177	BnaA01g24810D	RLK
			BnaA01g25640D	RLK
	C01	12,182,933	BnaC01g16720D	TM-CC
			BnaC01g18040D	NBS > NL
	C03	18,318,846	BnaC03g29930D	RLK
			BnaC03g30950D	RLK
	C03	23,396,866	BnaC03g35640D	TM-CC
			BnaC03g38180D	TM-CC
	C07	33,405,386	BnaC07g27490D	RLK
			BnaC07g28950D	RLP

## Discussion

Previous studies have shown that *Lm* populations evolve rapidly under the selection pressure from resistant canola/rapeseed cultivars carrying specific R genes [21, 22], and single R genes can be overcome due to the high diversity of *Lm* and a shift of pathogen population toward virulence [22]. Hence, it is critical for the canola industry to continue identifying novel alleles of resistance for sustainable management of blackleg. The GWAS allow us to rapidly identify and validate significant loci with associated markers. In this study, twelve associated regions were identified from *B. napus*, including 93 Canadian and 150 Chinese canola/rapeseed accessions, against 22 *Lm* isolates, under controlled-environment and/or field experiments. Some race nonspecific QTLs were identified in this study. For example, resistance-associated region on Chromosome A08 from Canadian varieties was effective against 15 of the 22 *Lm* isolates (Table S2 and Fig. 4). These significant regions with resistance to a broad range of *Lm* races are considered potential alleles with greater durability for blackleg resistance.

Three most significant resistance-associated regions, identified among Canadian accessions against 22 *Lm* isolates based on cotyledon inoculation, were on chromosomes A03, A08 and A09 (Table S2). However, the known R genes *Rlm1*, *Rlm2*, *Rlm3*, *Rlm4–7*, *Rlm6*, *LepR1*, *LepR2* or *LepR3* were not detected in these significant SNP loci based on the Avr genes carried by the Lm isolates used in the study. Our results show that the resistance alleles identified can be novel or they may interact with known R loci and produce new resistance specificity. Quantitative resistance has been identified on chromosomes A03, A08 and A09 previously [25–27, 29, 30, 34]. In this study, three associated SNP loci were found in a 572-kb region on chromosome A03 (24,158,622 to 24,701,214). One SNP and one QTL were identified also on chromosome A03 from studies of 179 Australia accessions [26] and linkage mapping of a “Darmor-*bzh*” x “Yudal” DH population [29], respectively, but these regions appear far away from the resistance-associated regions identified in the current study. Significant SNP loci or QTLs associated with cotyledon or adult-plant resistance have also been identified on chromosomes A08 and A09 using an association panel [30], DH [25, 27, 29] or F_2_/F_3_ Canola/rapeseed populations [34]. However, these loci were detected using Australian or French materials based on genetic-linkage maps with SSRs or other markers, and it is difficult to determine physical positions of these SNPs/QTLs precisely against the *B. napus* reference genome. In this study, we were able to narrow the range of resistance-associated SNP loci to an 11-kb region on chromosome A08 and 36 bp on chromosome A09, respectively, using GBS with *B. napus* reference genome for the resistance on cotyledons. This shows the advantage of using GWAS and GBS to identify resistance loci against blackleg disease of canola/rapeseed.

Five resistance-associated regions were located on chromosomes A08, C01, C04, C05 and C07 in the Chinese rapeseed accessions against 12 *Lm* isolates on cotyledons (Table S3). On chromosome A08, however, the physical position of the region appears different from that found with Canadian canola accessions. Although these regions have not been reported previously for *Lm* resistance at the seeding stage, the associated SNP loci were located in homologous regions, especially on chromosomes A01/C01, A04/C04/A05, C07/A03/C03. These regions may either reside functionally redundant loci or be involved in increased allelic diversity of the genes controlling the resistance to blackleg [33].

Our results showed that there were less number of QTLs against *L. maculans* identified from the Chinese accessions than the Canadian accessions, and also SNPs associated with cotyledon resistance to *L. maculans* were distributed in both A and C genomes of *B. napus* from the Chinese accessions, but mainly in the A genome from the Canadian accessions. One of the reasons for these could be due to different breeding focuses for resistance to canola diseases between the two countries. The selection and accumulation of canola against *L. maculans* have never been performed in canola breeding programs in China. Chinese accessions are winter-ecotype [64], and blackleg, caused by *L. maculans* has never been an issue in the canola growing areas in China. Hence, breeding for resistance to *L. maculans* has not been considered as one of the breeding objectives by Chinese canola breeders. On the other hand, *L. maculans* is an important disease on canola in western Canada. Great efforts have been made for breeding canola for resistance to blackleg in the past 3 years. Introgression of resistance genes derived from the A-genome species *B. rapa*, such as *LEM1* [65], *LmFr1* [66], *LmR1*/*CLmR1* [67, 68]*, Rlm1*, *Rlm3*, *Rlm4*, *Rlm7* and *Rlm9* [15, 66–69], and *LepR1* to *LepR4* [12–14] have been performed. Therefore, it would not be surprising that more QTLs were identified in the Canadian accessions and the resistance loci were mainly distributed in A genome of *B. napus*.

The completely sequenced and annotated genome of *B. napus* provides a useful reference to identify blackleg resistance candidate genes in canola/rapeseed germplasm pools. Plant resistance genes, such as CC-LRR-NBS, TIR-LRR-NBS, RLK, RLP and transmembrane proteins, can all be identified using the RGAugury pipeline [63]. Several potentially new R gene candidates were uncovered from the Canadian and Chinese canola/rapeseed accessions using genome-wide studies with the RGA pipeline, and these candidates have also been located close to the significant SNPs identified in GWAS, and can be potential blackleg-resistance genes supported by robust genetic and genomic analyses. They should be further explored for confirmation and applications. It is interesting to note that many of the well-known blackleg R genes, including *Rlm1*, *Rlm3* and *LepR3*/*Rlm2*, were not detected with GWAS in this study. This may be due to a lack of marker polymorphism in the mapping panel because of selected commercial varieties, such as the Canadian panel, has only moderate genome-wide coverage of makers and low frequencies of informative alleles (associated with blackleg resistance). Additionally, the Chinese panel was not selected originally for blackleg resistance, since the causal agent *Lm* had not been reported in China. The blackleg resistance loci identified in this study appear novel, and the study provides insights into several potentially new regions for discovery of additional blackleg R genes.

## Conclusion

In this study, twenty-two Lm isolates were used to identify resistance genes in a collection of 243 canola/rapeseed (*Brassica napus* L.) accessions from Canada and China. Thirty-two and 13 SNPs were identified from the Canadian and Chinese accessions, respectively, tightly associated with blackleg resistance with *P* values < 1 × 10–4 using a genome-wide association study (GWAS) and genotype-by-sequencing (GBS). These SNP loci were distributed on chromosomes A03, A05, A08, A09, C01, C04, C05, and C07, with the majority of them on A08 followed by A09 and A03. Our study provides insights into potentially new genomic regions for discovery of additional blackleg resistance genes. The identified regions associated with blackleg resistance in the germplasm collection may also contribute directly to the development of canola varieties with novel resistance genes against blackleg of canola.

### Plant source

Out of 150 Chinese *B. napus* accessions, 136 accessions were kindly provided by Prof. Baocheng Hu from Anhui Academy of Agricultural Sciences, Hefiei, Anhuhi, China, and 14 accessions were kindly provided by Prof. Yingze Niu from Sichuan Agricultural University, Chengdu, Sichuan Province, China.

## Methods

### Blackleg resistance evaluation using multiple *Lm* isolates

A diverse panel of 243 accessions of *B. napus*, including 93 Canadian canola varieties or breeding lines [22] and 150 Chinese canola varieties [60], was evaluated for seedling resistance against 22 *Lm* isolates with different compliments of Avr genes. Cotyledon inoculation assay was used to evaluate seedling resistance of 243 canola accessions under controlled environment (21 °C /16 °C with a 16 h photoperiod). Briefly, seven-day-old canola seedlings were punctured and inoculated with 10 μL *Lm* pycnidiospore suspension (2 × 10^7^ spores/mL). Lesion size on the cotyledons was scored 12–14 days post inoculation using the rating scale of 0–9 [22]. The average rating score (ARS)) was calculated from 48 inoculation sites. The interaction phenotype was evaluated as follows: ARS ≤ 4.5 was considered resistant reaction (R), ARS = 4.6–6.0 was intermediate reaction (I) and ARS = 6.1–9.0 was susceptible reaction (S) [22]. Details of Avr gene composition have been described previously for these isolates [22, 60]. All phenotypic data were performed on non-normalization and normalization anlysis based on previously published method [70].

### Reference-based SNP calling

Leaf-tissue samples were collected from true leaf of canola/rapeseed seedlings, freeze-dried in a Freezone 6 dryer (Labconco Corp, Kansas City, MO) for 48 h, then ground using the Mixture Mills 300 (Retsch Inc., Newtown, PA). DNA was isolated using a DNeasy 96 Plant Kit (Qiagen, Toronto, ON), quantified using a NanoVue Plus spectrophotometer (GE Healthcare, Piscataway, NJ), diluted to 10 ng μl^− 1^ and kept at − 20 °C until use in subsequent genotyping.

Raw 125-bp pair-end reads, with low-quality reads and adapters removed, were trimmed using Trimmomatic [71, 72] of the GBS-SNP-CROP pipeline V3.0 [61]. The filtered reads were aligned to the *B. napus* reference genome [62] using the Burrows-Wheeler Aligner program V0.7.12 [73] and GBS-SNP-CROP pipeline v3.0 [61]. For SNP calling and filtering, the GBS-SNP-CROP pipeline v3.0 [61] was used. SNPs and InDels were filtered with the following parameters: mnHoDepth0 = 11, mnHoDepth1 = 48, mnHetDepth = 3, altStrength = 0.9, mnAlleleRatio = 0.1, mnCall = 0.75, mnAvgDepth = 3 and mxAvgDepth = 200.

### Population structure (Q) and kinship coefficients (K)

To determine the Q value of population structure accurately, STRUCTURE 2.3.3 [74] (http://pritch.bsd.uchicago.edu/software.html) was performed with 503 selected SNPs, which were evenly distributed with 5 Mb intervals on each chromosome of *B. napus*. In STRUCTURE 2.3.3, the admixture ancestry and independent allele frequency parameters were used in the model with a burn-in period of 100,000 and a Markov chain Monte Carlo Model (MCMC) with 100,000 repetitions as suggested by Pritchard and Wen [75]. It was assumed that sub-populations (K) were set to 1–10, with 20 independent iterations run for each K. The obtained *Q*-matrices were used to assign *B. napus* lines to defined sub-populations. Structure Harvester v6.7 [76] was used to determine the number of sub-populations according to Evanno et al. [77]. Finally, the Q-matrices were determined with CLUMPP (V1.1.2b) [78].

The population structure was verified by PCA using kinship coefficients among lines with SNP and InDel genotypic data (filtered for 5% minor allele frequency) for the 243 canola/rapeseed lines in TASSEL 5.0 [79].

### Genome-wide association studies

The association of SNP and InDel markers with blackleg resistance was examined in TASSEL 5.0 [79]; all markers were filtered for 5% minor allele frequency (MAF) and 10% missing genotypes and the filtered genotyping data were used for association analysis. The kinship matrix (K) and Principal Component Analysis (PCA) were estimated using filtered SNPs in TASSEL 5.0. Both general linear models (GLM) (GLM + Q and GLM + PCA) and mixed linear models (MLM) (MLM + Q + K and MLM + PCA + K) were performed to investigate best fit models in the current study. Additional, original and normalized phenotypic data were ananlyzed using the above four model. A positive false discovery rate (pFDR) of 5% (*q* < 0.05) [80] was applied to test the statistical significance of all detected SNP loci. The phenotypic traits of SNP marker alleles were calculated as the difference between the mean phenotypes of the two marker classes (presence or absence of marker alleles) in TASSEL 5.0. RGA candidates were identified with the RGAugury pipeline [63] in the *B. napus* reference genome [62].

## Supplementary information

**Additional file 1.**

**Additional file 2.**

**Additional file 3.**

**Additional file 4.**

**Additional file 5.**

**Additional file 6.**

## Data Availability

The datasets used and/or analyzed during the current study available from the corresponding authors on reasonable request.

## Abbreviations

CV: Coefficient of variation
GBS: Genotype-by-sequencing
FDRs: False discovery rates
GLM: General linear model
GWAS: Genome-wide association study
*Lm*: *Leptosphaeria maculans*
RGAs: Resistance gene analogues
LOD: Logarithm of odds
RLKs: Receptor-like kinases
RLPs: Receptor-like proteins
TM-CC: Transmembrane coiled-coil proteins
AM: Association mapping
MAS: Marker-assisted selection
QR: Quantitative resistance
ETI: Effector-triggered immunity
PG: Pathogenicity group (PG)
NBS: Nucleotide binding site
MLM: Mixed linear model
PCA: Principal component analysis
QTL: Quantitative trait loci
SNP: Single nucleotide polymorphism
