# Supplementary figures and images for: Identification of resistance loci in Chinese and Canadian canola/rapeseed varieties against Leptosphaeria maculans based on genome-wide association studies
Source: BMC Genomics. 2020 Jul 21;21:501. doi: 10.1186/s12864-020-06893-4 (PMC7372758; doi:10.1186/s12864-020-06893-4)

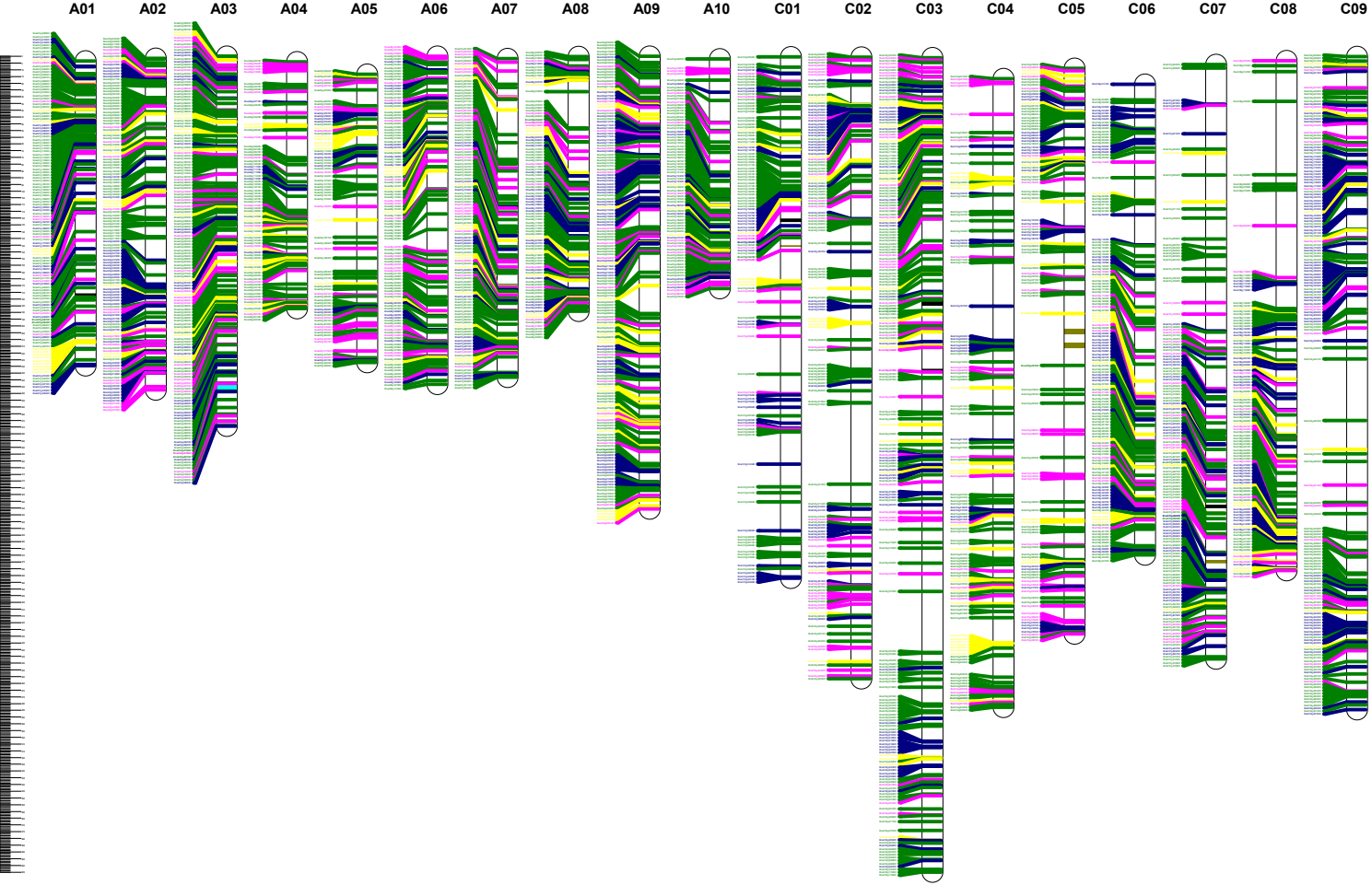

Supplement: Supplementary file 1 — Additional file 1. [file 12864_2020_6893_MOESM1_ESM.pdf]
